# Three-dimensionally preserved appendages of the early Cambrian trilobite *Yunnanocephalus yunnanensis*

**DOI:** 10.7717/peerj.21095

**Published:** 2026-06-25

**Authors:** Luis Collantes, Sarah R. Losso, Xianguang Hou, Huijuan Mai, Michel Schmidt, Yu Liu

**Affiliations:** 1 Yunnan Key Laboratory for Palaeobiology, Institute of Paleontology, Yunnan University, Kunming, China; 2 MEC International Joint Laboratory for Palaeobiology and Palaeoenvironment, Yunnan University, Kunming, China; 3 Museum of Comparative Zoology and Department of Organismic and Evolutionary Biology, Harvard University, Cambridge, United States of America; 4 Bavarian State Collection of Zoology, Bavarian Natural History Collections, Munich, Germany; 5 School of Geography, Geology and the Environment, University of Leicester, Leicester, United Kingdom

**Keywords:** Trilobita, X-ray microcomputed tomography, Ventral anatomy, Chengjiang biota

## Abstract

Advances in micro-computed tomography have brought new insights into the non-biomineralized three-dimensional morphologies of various euarthropods from the Chengjiang biota. The application of this technique to *Yunnanocephalus yunnanensis,* as described herein, also helps reveal the well-preserved 3D morphologies of this early Cambrian trilobite for the first time. The two study specimens bear a pair of uniramous antennae, four post-antennal cephalic appendages, and up to sixteen thoracic appendages. The first post-antennal appendage has a well-developed endopodite and exopodite, both diverging from a protopodite—an organization that could aid in respiration, feeding, and locomotion, but has not been known in any other Cambrian redlichiids. All the following biramous appendages consist of a protopodite transversely elongated into a robust gnathobase, a bipartite exopodite with sagittally broad podomeres bearing dumbbell-shaped marginal lamellae, and an endopodite composed of seven endite-bearing podomeres that become more elongated distally. The present study not only adds to the diversity of appendage morphologies in redlichiids but also helps understand how trilobites may have survived under different selective pressures by “upgrading” their feeding, sensory, and locomotory apparatus.

## Introduction

In the last two decades, our knowledge about the ventral anatomy of trilobites has substantially increased (*e.g.*, Hou et al., 2008; Gutiérrez-Marco et al., 2017; Zeng et al., 2017; Holmes, Paterson & García-Bellido, 2020; Budil & Fatka, 2021; Siveter et al., 2021; Hou, Hughes & Hopkins, 2021; Hou, Hughes & Hopkins, 2023; Hou et al., 2023; Pérez-Peris et al., 2021; El Albani et al., 2024; Losso & Ortega-Hernández, 2022; Losso & Ortega-Hernández, 2024; Losso, Thines & Ortega-Hernández, 2023; Losso et al., 2023). This improvement is due, among other factors, to the use of new methodologies such as X-ray microcomputed tomography, which provides unprecedented resolution for the visualization and description of the trilobite appendages (*e.g.*, Hou, Hughes & Hopkins, 2021; El Albani et al., 2024). To date, non-biomineralized anatomy (including antennae, biramous appendages, and digestive tract) has been found in more than 40 species of trilobites. Among these, only ca. 20 species have been reported to preserve all or part of the appendages (see Losso & Ortega-Hernández, 2022). Despite the abundance of trilobites, with more than 4,000 described genera and 22,000 species, only 0.5% are preserved with appendages, and 0.1% with relatively complete biramous appendages (Jell & Adrain, 2003; Adrain, 2008; Paterson, 2020).

The early Cambrian Chengjiang biota from Yunnan province, southwestern China, has emerged as a global reference point because of the abundance of Cambrian metazoans with exceptional soft-tissue preservation (Hou et al., 2004; Hou et al., 2017). However, as mentioned in previous works (Hou, Ramsköld & Bergström, 1991; Hou et al., 2004; Chen & Zhuo, 1997), trilobites do not represent a dominant group in comparison with other euarthropods from this biota. Among trilobites, four genera have been described from the Chengjiang deposits: *Eoredlichia* Chang in Lu & Dong, 1952, *Yunnanocephalus* Kobayashi, 1936, *Kuanyangia* Hupé, 1953, and *Wutingaspis* Kobayashi, 1944, of which the first three are preserved with appendages (Shu et al., 1995; Hou & Bergström, 1997; Hou et al., 2008). The ventral anatomy of these trilobites has received little attention compared to other Chengjiang euarthropods (Shu et al., 1995; Ramsköld & Edgecombe, 1996; Hou et al., 2008). In addition, these studies have based their descriptions and line drawings on direct observation of the heavily compressed fossils, which hinders the morphological reconstruction of the appendicular structure of trilobites, rather than utilizing micro-computed tomography (micro-CT), which has elucidated the ventral anatomy of other euarthropods (especially artiopodans) from Chengjiang (*e.g.*, Chen et al., 2019; Zhai et al., 2019; Zhang et al., 2022; Zhang et al., 2024; Schmidt et al., 2022).

In the present study, we describe the three-dimensional ventral anatomy of the trilobite *Yunnanocephalus yunnanensis* (Mansuy, 1912) for the first time, by means of micro-CT and computer-based 3D rendering. *Yunnanocephalus* represents the oldest trilobite species to show three-dimensional ventral anatomy with this technique to date. Notably, the first post-antennal appendage of *Yunnanocephalus* is biramous with well-developed exopodite and endopodite, unlike those seen in other Cambrian redlichiids from the Tatelt Formation (El Albani et al., 2024) indicating variability in ecological roles.

## Materials and Methods

### Material

Specimens are housed in the Yunnan Key Laboratory for Palaeobiology, Institute of Palaeontology, Yunnan University (YKLP). The studied material consists of two specimens of *Yunnanocephalus yunnanensis* (YKLP 17210 and YKLP 17211) (see Supplementary material 1). Four specimens were scanned in total, but only two preserved ventral anatomy. *Y*. *yunnanensis* is a relatively common species of the Chengjiang biota, distributed along multiple fossil sites of the Yunnan province (Steiner, Weber & Geyer, 2001). The specimens were collected from the Yu’anshan Member, Chiungchussu Formation, *Eoredlichia*–*Wutingaspis* trilobite biozone, Cambrian Series 2, Stage 3, Mafang village, Haikou county, Kunming, Yunnan, China.

Fossils from the Chengjiang biota were rapidly buried in a clay-rich sediment and pyritized during early diagenesis, which later oxidized into pseudomorphs (Gabbot et al., 2004). This type of preservation provides the conservation of non-biomineralized anatomy, such as the appendages or the digestive tract, as well as fossil-matrix density differential needed for the application of X-ray microcomputed tomography.

### Descriptive terminology

We follow the standard terminology for trilobites in Whittington & Kelly (1997), including ‘antenna(e)’, ‘endopodite(s)’, and ‘exopodite(s)’. The term ‘article(s)’ is used for divisions of the antenna, and ‘podomere(s)’ for divisions of the endopodite and exopodite. Podomeres are counted from distal to proximal, with one being the claw and seven being the proximal-most following Bruce & Patel (2020) and Bruce (2021). Appendages are divided between antennae (an), post-antennal cephalic appendages (C), and thoracic appendages (T). Appendages of the right side (dorsal view) are indicated by the letter “r”, while appendages from the left side are indicated with the letter “l”.

### Micro-computed tomography and 3D rendering

The specimens were scanned with Xradia 520 Versa (Carl Zeiss X-ray Microscopy, Inc., Pleasanton, USA) at Yunnan University. Scanning parameters are as follows: **YKLP 17210**: Beam strength: 50kV/4W, no Filter, Pixel size: 9.92 µm, Number of TIFF images: 2558; **YKLP 17211**: Beam strength: 50kV/4W, no Filter, Pixel size: 11.91 µm, Number of TIFF images: 2212; ROI scans of the left and right parts of the cephalon in **YKLP 17211**: Beam strength: 80kV/7W, no Filter, Pixel size: 6.21 µm, Number of TIFF images: 1656; Beam strength: 80kV/7W, no Filter, Pixel size: 5.58 µm, Number of TIFF images: 1729. The 3D rendering and segmentation was performed using Drishti 2.4 (Limaye, 2012), and figures were arranged with Adobe Photoshop 2021. Three-dimensional interpretative reconstruction of the appendage of *Y*. *yunnanensis* was made in Blender software v5.0.1.

## Results

### General features of appendages

**Figure 1:**
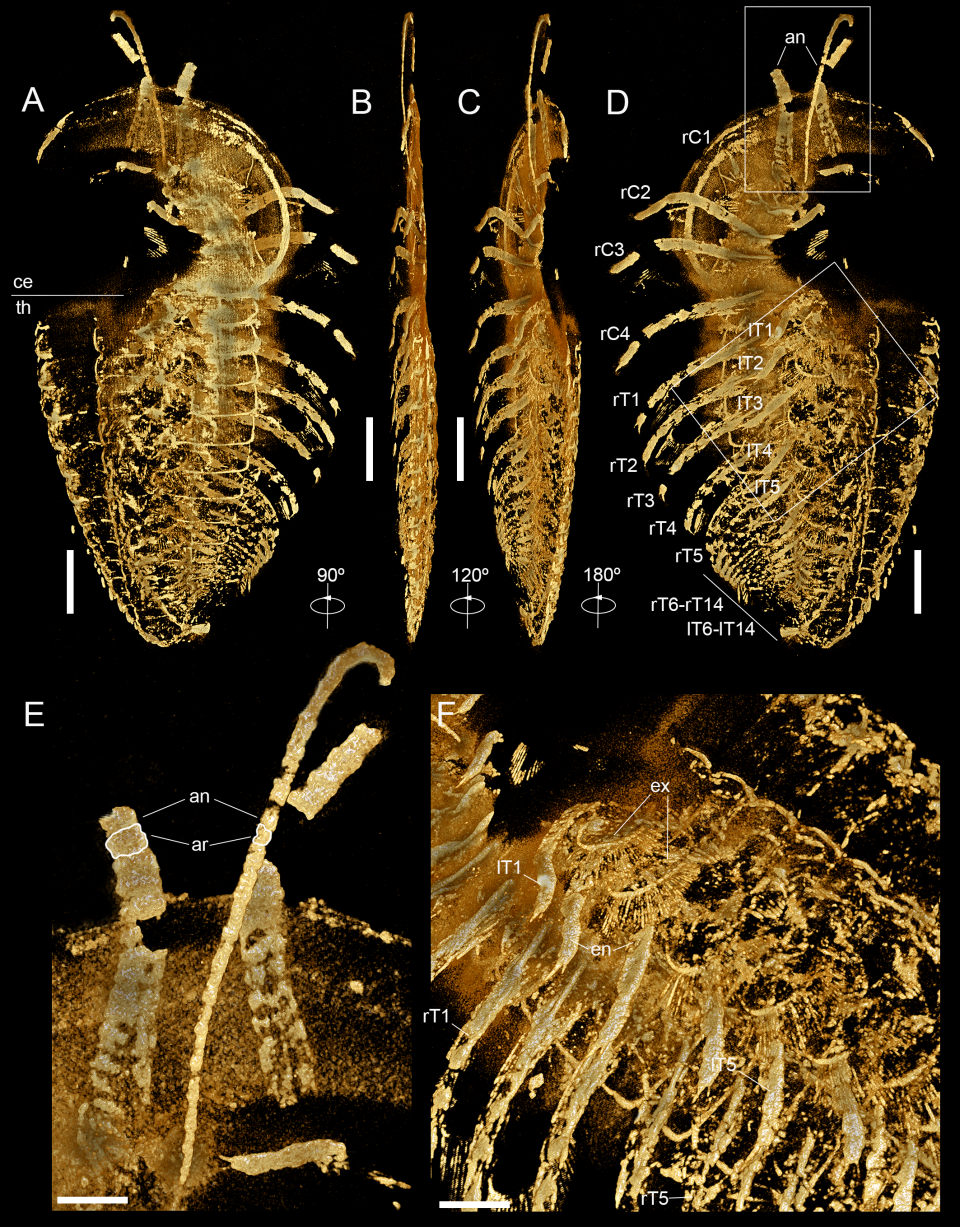
X-ray microcomputed tomography model of *Yunnanocephalus yunnanensis* (Mansuy, 1912), specimen YKLP 17210. (A) Dorsal view. (B) Lateral view. (C) Ventral-lateral view. (D) Ventral view. (E) Detail of the antennae. (F) Detail of the first thoracic appendages in ventral view. Abbreviations: an, antennae; ar, articles; ce, cephalon; en, endopodite; ex, exopodite; lC1–lC4, left cephalic appendages; lT1–lT14, left thoracic appendages; rT1–rT14, right thoracic appendages; th, thorax. Scale bars: two mm (A–D), 0.5 mm (E), one mm (F).

**Figure 2:**
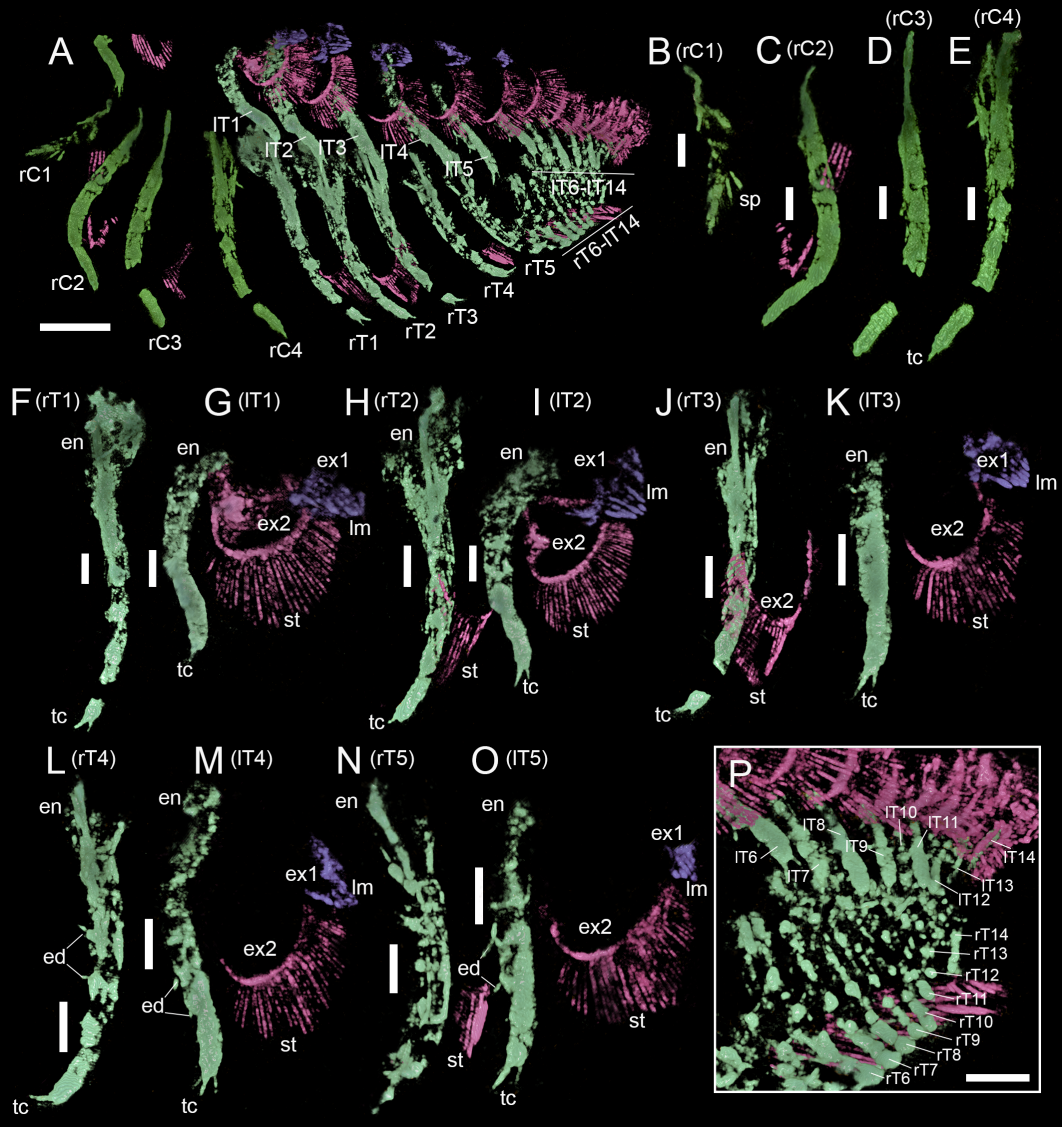
Segmentation of the different appendages of *Yunnanocephalus yunnanensis* (Mansuy, 1912), specimen YKLP 17210. (A) General view of the ventral anatomy. (B –E) Post-antennal cephalic appendages. (B) rC1, ventral view. (C) rC2, dorsal view. (D) rC3, dorsal view. (E) rC4, dorsal view. (F–O) Thoracic appendages. (F) rT1, dorsal view. (G) lT1, ventral view. (H) rT2, dorsal view. (I) lT2, ventral view. (J) rT3, dorsal view. (K) lT3, ventral view. (L) rT4, dorsal view. (M) lT4, ventral view. (N) rT5, dorsal view. (O) lT5, ventral view. (P) Detail of the posterior-most thoracic appendages. Abbreviations: ed, endites; en, endopodite; ex1, exopodite (proximal lobe); ex2, exopodites (distal lobe); lm, lamellae; lT1–lT14, left thoracic appendages; rC1–rC4, right cephalic appendages; rT1–rt14, right thoracic appendages; sp, spines; st, setae; tc, terminal claw. Scale bars: two mm (A), 0.5 mm (B–P).

**Figure 3:**
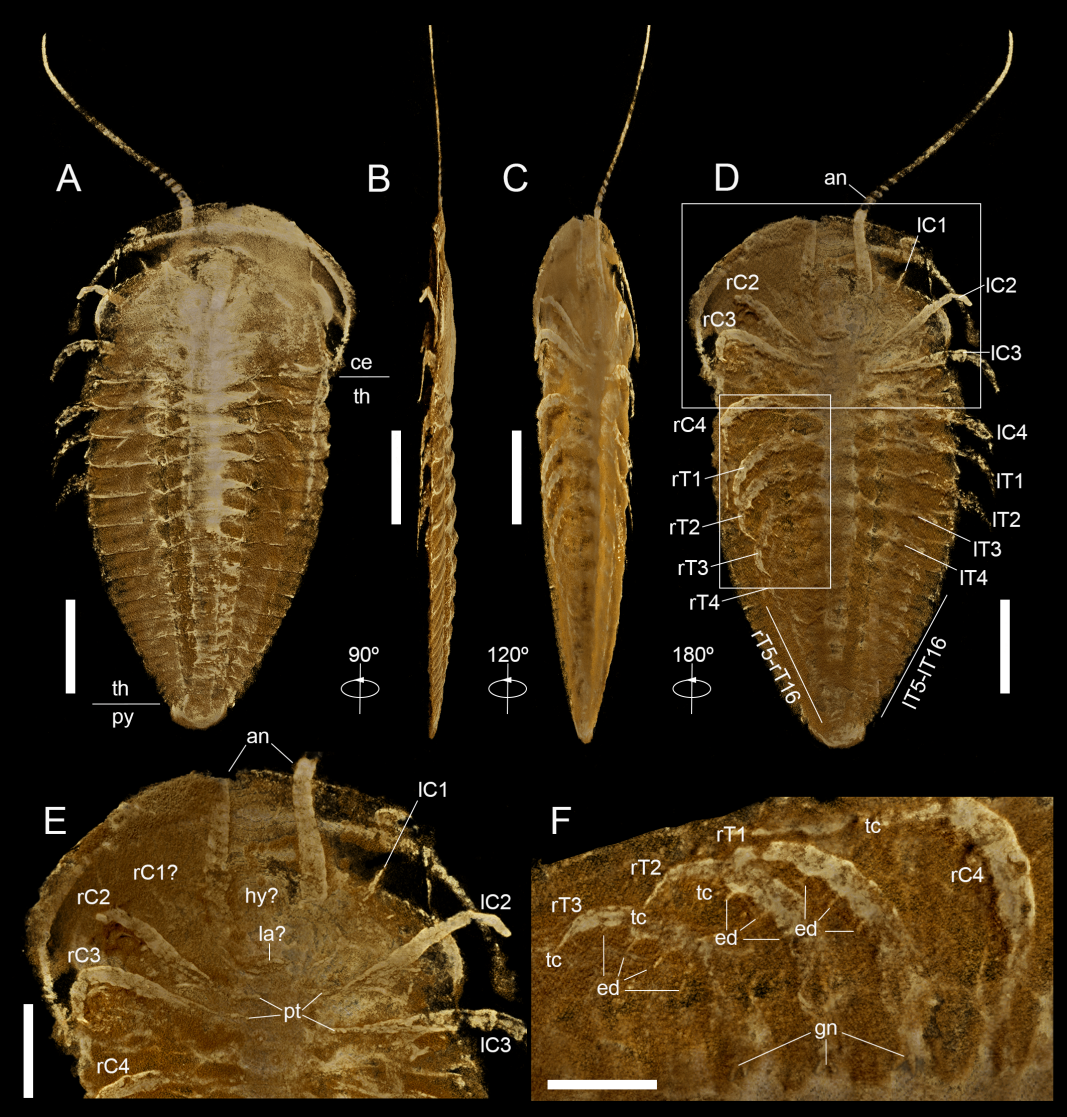
X-ray microcomputed tomography model of *Yunnanocephalus yunnanensis* (Mansuy, 1912), specimen YKLP 17211. (A) Dorsal view. (B) Lateral view. (C) Ventral-lateral view. (D) Ventral view. (E) Ventral view of the cephalic area. (F) Detail of the first thoracic appendages in ventral view. Abbreviations: an, antennae; ed, endites; en, endopodite; ex, exopodite; gn, gnathobasic spines; hy?, putative hypostome; la?, putative labrum; lm, lamellae; lC1–lC4, left cephalic appendages; lT1–lT16, left thoracic appendages; rC2–rC4, right cephalic appendages; rT1–rT16, right thoracic appendages; tc, terminal claw. Scale bars: three mm (A–D), two mm (E–F).

**Figure 4:**
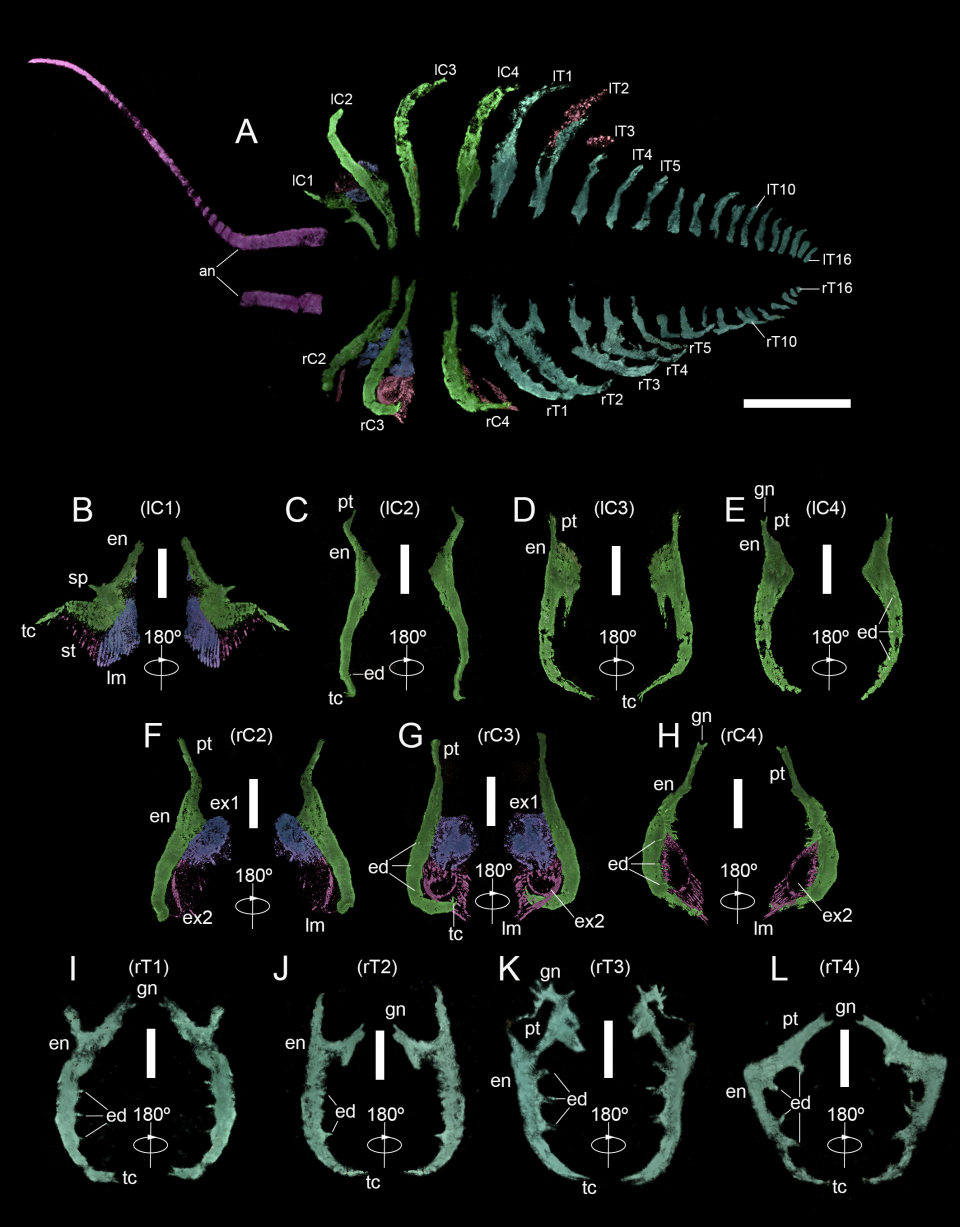
Segmentation of the most informative appendages of *Yunnanocephalus yunnanensis* (Mansuy, 1912), specimen YKLP 17211. (A) General view of the ventral anatomy. (B–H) Post-antennal cephalic appendages. (B) lC1, ventral view and dorsal view. (C) lC2, ventral and dorsal view. (D) lC3, ventral and dorsal view. (E) lC4, ventral and dorsal view. (F) rC2, dorsal and ventral view. (G) rC3, dorsal and ventral view. (H) rC4, dorsal and ventral view. (I–L) Selected thoracic appendages, (I) rT1, dorsal and ventral view. (J) rT2, dorsal and ventral view. (K) rT3, dorsal and ventral view. (L) rT4, dorsal and ventral view. Abbreviations: an, antennae; ed, endites; en, endopodite; ex1, exopodite (proximal lobe); ex2, exopodite (distal lobe); lC1–lC4, left cephalic appendages; lm, lamellae; lT1–lT14, left thoracic appendages; pt, protopodite; rC2–rC4, right cephalic appendages; rT1–rT14, right thoracic appendages; sp, spines; st, setae; tc, terminal claw. Scale bars: three mm (A), one mm (B–L).

*Yunnanocephalus yunnanensis* has a single pair of uniramous antennae (an), followed by a series of post-antennal biramous limbs, divided into four cephalic (rC1–rC4) appendages and up to sixteen thoracic appendages (rT1–rT14, lT1–lT14) (Figs. 1–2). Only 14 thoracic appendage pairs are visible in YKLP 17210 (Figs. 1–2), with well-preserved endopodites of rC1–rC4, and incomplete exopodites in rC2 and rC3. Appendages increase in size posteriorly until lC4 and then decrease along the trunk. In YKLP 17211 (Figs. 3–4) the endopodites of rC2–rC4 and lC1–lC4 are well-preserved, but exopodites are only visible in lC1, lC3, and rC2–rC4. Thoracic endopodites are well preserved, while the exopodites are overlapping with each other and are not clearly recognizable.

### Antennae

The paired uniramous antennae (an) are presumably attached dorsal to the anterior part of the hypostome in YKLP 17211 (Fig. 3D). Attachment between the antenna and inner ventral side of the cephalic shield is unclear (Fig. 1E). The length of the complete extended antennae is 9.5 mm in YKLP 17210 to 11 mm in YKLP 17211 and occupies an extension that is almost twice the length (sag.) of the cephalon. Individual antennae may be curved by up to 30° from the sagittal axis (dorsal and ventral view) at the anterior margin of the cephalon (Figs. 1E, 4A). The number of articles preserved goes from 36 in YKLP 17210 to 45 articles in YKLP 17211, although the maximum number of articles cannot be determined. The most proximal articles are stout, narrowing distally, subrectangular to trapezoidal in the basal part, and turning cylindrical distally (Figs. 1E, 3D).

### Post-antennal appendages

Appendage rC1–lC1 shows a distinct morphology from the posterior cephalic appendages (Figs. 1E, 4B). It originates anterodorsal to the posterior margin of the hypostome (Fig. 3E). The hypostome is ovoidal to subrectangular in YKLP 17211, with a maximum sagittal length of 2.0 mm from the anterior margin to the posterior one, and a maximum width of 1.1 mm at the insertion of the antennae (Fig. 3E). A lobate projection in the posterior margin of the hypostome is identified as a putative labrum (Fig. 3E). The protopodite is preserved in YKLP 17211, it is sagittally narrow and exhibits a subtle curvature anteriorly. After this curvature, the endopodite extends towards the anterior cephalic border. The endopodite is composed of seven podomeres, including the terminal claw. The proximal portion of the endopodite projects posteriorly in ventral view (Fig. 2A). The articulation between podomeres 2 and 3 is angled, with the terminal claw extending towards the anterior cephalic border. Podomeres are rectangular in outline, becoming narrower and shorter distally (Figs. 2B, 4B). Podomere 4 bears two large, diverging spines near the distal margin (Figs. 2B, 4B). Podomere 5 bears a line of small, straight lateral spines in YKLP 17210 (Fig. 2B).

Appendages rC2–lC2 and rC3–lC3 show similar dimensions and morphology (Figs. 4C–4D, 4F–4G). They presumably originate dorsal to the hypostome. The protopodite is thin and anterior-posteriorly flattened in YKLP 17211 (Figs. 4C–4D, 4F–4G). The proximal-most part of the endopodite is antero-posteriorly compressed (Figs. 2C, 2D). Both endopodites show a marked curvature starting in the distal third of the endopodite. Six podomeres are visible. The podomeres are cylindrical with a rectangular outline, elongating distally. Terminal claw composed of three short, thin claws, preserved in lC3 of YKLP 17211 (Fig. 4D). Appendages lC2 and lC3 of YKLP 17211 preserve fragments of the exopodite, while appendage rC3 shows an almost complete exopodite, although the lamellae are not clearly distinguishable (Fig. 4G). The exopodite overall morphology is ovoid in outline, with a thick, strongly curved lateral margin. The short axis of the ovoid represents 3/5 of the long axis. Lamellae fan out from the ovoid margin with radial display. The exact number and disposition of the lamellae are not clear. Setae are long and thin on the distal lobe. Each seta extends between 0.4–0.7 mm.

Appendages rC4–lC4 originates close to the junction of the cephalon with the thorax, dorso-posterior to the hypostome (Figs. 4E, 2H). Both are slightly longer than rC2–lC2 and rC3–lC3. The protopodite is transversely elongated and anterior-posteriorly flattened, but the shape in anterior view is unclear (Figs. 4E–4H). The endopodite is more robust than the anterior appendages. Both appendages show a clear segmentation of the six podomeres, with clear podomere boundaries. Proximal podomeres are similar in width until podomere 5, then progressively narrow distally. Three to four pairs of small endites are present along the endopodite, both medially and distally, from podomere 3 to 6, preserved as short, robust spines (Fig. 4H).

Appendages rT1–rT5/lT1–lT5 show similar morphology and proportions, whereas posterior limbs become progressively smaller posteriorly (Figs. 2A, 4A). Each endopodite bears six podomeres plus a terminal piece composed of three terminal claws (Fig. 2L). Podomeres are thicker proximally and narrower distally. Endopodites show a smooth curvature, beginning in the joint of the first and second podomere, with convexity to the ventral side, extending homogeneously to the proximal end (Figs. 2F–2O, 4I–4L). The dorsal margin of rT3 protopodite in YKLP 17211 is poorly preserved, but a robust gnathobase is present at the ventral edge of the medial margin (Figs. 3F, 4K). The medial margin appears to be angled dorsolaterally and fairly transversely broad (Figs. 4I–4L). Each endopodite bears six podomeres plus a terminal piece composed of three terminal claws. The podomere outline is sub-rectangular to trapezoidal. Podomeres are thicker proximally and progressively narrow distally. Subtle curvature takes place between the fourth and fifth podomere on all endopodites, with convexity to the dorsal margin. Three to four pairs of short, stout endites are present along the endopodite, both medially and distally, from podomere 3 to 6, preserved as short, robust spines (Figs. 2L–2O, 4I–4L). The endopodite terminates in a tripartite terminal claw (Fig. 4K). From rT6–lT6 to rT16–lT16, endopodites are similar in morphology but decrease in size posteriorly (Fig. 4A).

Exopodite structure is clearly observed in lT1–lT5 from YKLP 17211, while those of lT6–lT14 overlap with each other. On the other side, in rT2, rT3, and rT5, only the distal part of the exopodite is preserved. The exopodites of lT1 to lT5 are ovoid in outline, with a thick, strongly curved margin. The short axis of the ovoid represents 3/5 of the long axis. Lamellae on the proximal lobe are thick, closely spaced, and relatively short, between 0.3 and 0.5 mm (Figs. 2H, 2J). Setae surrounding the distal lobe are long, thin, and broadly splayed. Each exopodite from lT1 to lT5 bears at least 14 lamellae and between 28–36 setae. Each seta extends between 0.3–0.6 mm, being longer in the middle ones (Figs. 2F–2O). Exopodites from lT6 to lT14 show similar morphology but progressively reduce towards the posterior area.

## Discussion

**Figure 5:**
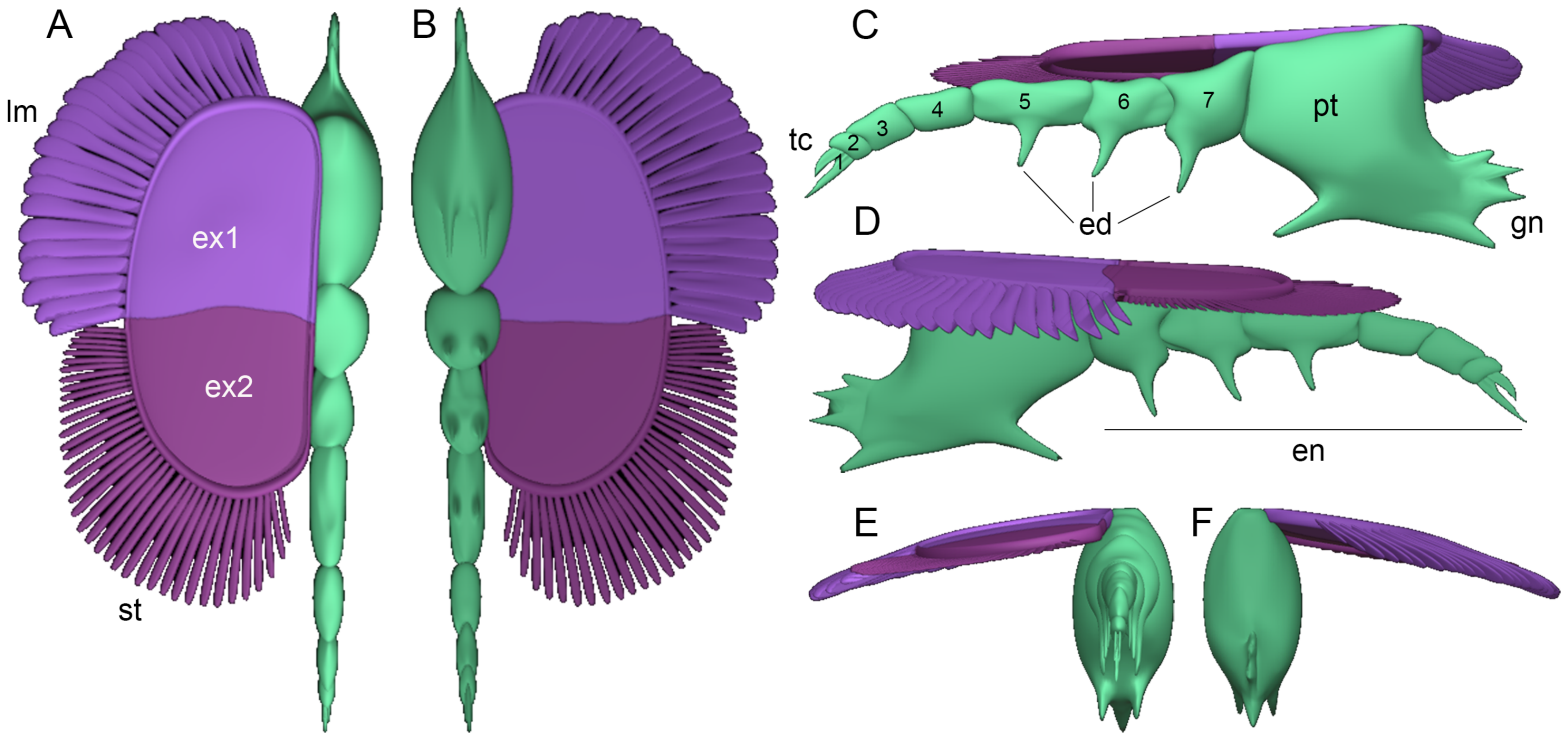
Three-dimensional reconstruction of the biramous appendage of *Yunnanocephalus yunnanensis* (Mansuy, 1912). (A) Dorsal view. (B) Ventral view. (C) Anterior view. (D) Posterior view. (E) Lateral (distal) view. (F) lateral (proximal) view. Morphology of the protopodite and all the characters along the endopodite are based on the direct observation of the scans of the studied specimens, while the overall morphology of the exopodite has been reconstructed by inference based on the partially preserved exopodites of the two specimens. Abbreviations: ed, endites; en, endopodite; ex1, exopodite (proximal lobe); ex2, exopodite (distal lobe); gn, gnathobasic spines; lm, lamellae; pt, protopodite; st, setae; tc, terminal claw. Not to scale.

Our micro-CT scans of *Yunnanocephalus yunnanensis* allow for a more in-depth understanding of the ventral non-biomineralized morphology. All the discussed observations are based on the studied specimens only, but they may not represent species-level characters accurately. The broad exopodite podomere morphology seen in *Y. yunnanensis* (Fig. 5) is similar to other redlichiid trilobites like *Hongshiyanaspis yiliangensis* from the Hongjingshao Formation (Zeng et al., 2017) and *Redlichia rex* from Emu Bay (Holmes, Paterson & García-Bellido, 2020). This morphology has been suggested as enhancing its capability for swimming, as well as potentially improving respiratory efficiency (Zeng et al., 2017). However, further investigation to test this hypothesis have not been conducted. A benthic lifestyle (as presumed for *Y*. *yunnanensis*) would likely require either negative lift or some stabilizing mechanism to prevent the animal from being lifted off the seafloor, which has been discussed by means of fluid dynamics (*e.g.*, Esteve et al., 2021). In addition, other features such as the overall body shape or the dorsal exoskeleton should also be taken into consideration for these interpretations. The protopodites appear to be wedge-shaped, tapering ventrally as seen in *Flexicalymene senaria* and *Ceraurus pleurexanthemus* (Losso, Thines & Ortega-Hernández, 2023), indicating this adaptation for enrolment appeared in the Cambrian.

### Endite morphology

All known trilobite endopodites have a similar overall morphology, composed of six podomeres and a terminal claw (Brunton & Haas, 1999; Hou et al., 2008; Gutiérrez-Marco et al., 2017; Zeng et al., 2017; Holmes, Paterson & García-Bellido, 2020; Hou, Hughes & Hopkins, 2021; Siveter et al., 2021; Pérez-Peris et al., 2021; Losso & Ortega-Hernández, 2024; El Albani et al., 2024). The major differences between the endopodites of trilobite species lie in the general outline, dimensions of the podomeres, and the presence or absence of endites. Endites of the studied specimens of *Y*. *yunnanensis* are in pairs, long and stout, with homogeneous distribution along the endopodite (Figs. 2M–2N, 4I–4L). Other Cambrian trilobites, such as *Eoredlichia intermediata*, *Olenoides serratus* or *Hongshiyanaspis yiliangensis,* bear similar spines, with the difference that the endites are grouped into clusters of two to four spines, and generally the endites are more developed in the proximal part of the endopodite of these species (see Whittington, 1980, pl. 20, figs. 1–4, pl. 21, figs. 1–3; Hou et al., 2008, fig. 7F–G; Zeng et al., 2017, figs. 12–13). Other post-Cambrian trilobites, such as *Ceraurus pleurexanthemus* or *Chotecops ferdinandi*, also bear long endites but with a delicate structure, with greater development in the proximal podomeres (see Losso & Ortega-Hernández, 2024, fig. 3B, 4B; Brunton & Haas, 1999, text-fig. 8, pl. 8). However, given the limited number of specimens studied in the present work, we cannot state unequivocally that this distribution of the endites along the endopodites of *Y*. *yunnanensis* corresponds with a taxonomical character or with a preservational artifact.

### First cephalic appendage in trilobites

The first post-antennal cephalic appendage (C1) is rarely preserved in trilobites, with only clear examples in *Protolenus (Hupeolenus)* sp. and *Gigoutella mauretanica* from the Cambrian Tatelt Formation (El Albani et al., 2024), and less well-preserved in *Triarthrus eatoni* from the Upper Ordovician of New York (Hou & Hopkins, 2024). Notably, in the first two examples, the protopodite is similar in shape to the posterior appendages, but the endopodite is absent or greatly reduced, while the exopodite is flagelliform (El Albani et al., 2024, fig. 3E–F). This contrasts with the first appendage seen in *Yunnanocephalus yunnanensis,* where both the endopodite and exopodite are well developed (Fig. 4B). Putative first cephalic post-antennal appendages are also observed in impressions of *Eoredlichia intermediata* (Hou et al., 2008, fig. 2A, 3), consisting of a flattened exopodite, and in *Hongshiyanaspis yiliangensis* (Zeng et al., 2017, figs. 4e, 6), consisting of a large, stout endopodite with a clear division of the podomeres. This variation in the nature of the first cephalic appendage shows a broad diversity within Redlichiida so far unseen in later trilobites. In later examples, such as *Triarthrus eatoni* from the Ordovician or *Chotecops ferdinandi* from the Devonian, the preserved first cephalic post-antennal appendages are limited to partially preserved, well-developed endopodites (Hou & Hopkins, 2024, figs. 1–3; Brunton & Haas, 1999, figs. 1, 4–7).

High fidelity three-dimensional preservation as is seen in *Y. yunnanensis, Protolenus (Hupeolenus)* and *Gigoutella* is crucial in providing insights into the first post-antennal appendage. Although preserved through different pathways, entombment in volcanic clastic sediment leaving a 3D void pace in the Tatelt Formation (El Albani et al., 2024) compared to the pyritized fossils of the Chengjiang biota, both provide high density contrast and retain fine details such as lamellae and endites. The variation in first post-antennal appendage between these three taxa is unlikely to result from preservation differences.

Sensory (tactile) functions in the first post-antennal appendage are inferred to be used in *Sinoburius lunaris* Hou, Ramsköld & Bergström, 1991 given the presence of derived anterior appendages replacing the antennae (see Chen et al., 2019), and likely for the flagelliform exopodites seen in *Protolenus (Hupeolenus)* and *Gigoutella* (El Albani et al., 2024). Endopodites are interpreted for locomotion and grasping food to bring it towards the mouth (Esteve & Rubio, 2023; Losso et al., 2025), whereas the protopodites were used to masticate food (Bicknell et al., 2021). Reduction of the endopodite would require the posterior appendages to grasp food, but the large protopodite seen in *Protolenus (Hupeolenus)* and *Gigoutella* could still masticate food. The first post-antennal appendage in *Y. yunnanensis* does not seem to have features specialized for sensing the environment, but likely played roles in respiration, swimming, grasping food, and mastication. The morphology of first post-antennal appendage may reflect features that evolved independently compared to the exoskeleton as is seen with cephalic structures (Holmes, 2023). Additionally, Park & Kihm (2017) noted that redlichiids exhibit variable conditions in the frontal glabellar region, whereas in other trilobite groups the glabellar structure is rather constant, suggesting possible modifications in feeding strategy during redlichiid evolution. Phylogenetic analyses by Paterson, Edgecombe & Lee (2019) recovered *Protolenus* as sister to *Hamatolenus* in a clade with *Strenuaeva* and *Ellipsocephalus*, with *Yunnanocephalus* branching just basal to this group. Better understanding of the function, diversity and evolution of the first post-antennal cephalic appendage requires additional work on the relationships between large clades and discovery of more taxa with this structure preserved.

### Limitations of the study

The description of the ventral anatomy of *Y*. *yunnanensis*, and the interpretations made based on it, are solely based on the scans of two specimens, which can be considered a small sample size. Four specimens of *Y*. *yunnanensis* have been scanned, but only the two specimens presented in this paper have provided elements of ventral anatomy (see Material). Therefore, the limited number of specimens has implications for how far the results can be generalized at the species level. However, the descriptions based on these three-dimensional models offer a very accurate picture of the appendages of this trilobite species. Furthermore, the fossil record of trilobites with preserved appendages is very scarce, which greatly limits anatomical comparisons between species.

## Conclusions

The application of X-ray micro-computed tomography to *Yunnanocephalus yunnanensis* provides the first three-dimensional reconstruction of the ventral anatomy of this early Cambrian trilobite and represents the oldest trilobite taxon studied with this technique to date. These data significantly expand our understanding of appendage organization and functional diversity within early Redlichiida.

The studied specimens reveal a limb series consisting of uniramous antennae followed by four post-antennal cephalic appendages and up to sixteen thoracic appendages. Particularly notable is the morphology of the first post-antennal appendage, which bears both a well-developed endopodite and exopodite. This condition contrasts sharply with the reduced or specialized first post-antennal appendages reported in other Cambrian redlichiids and suggests a previously undocumented functional versatility in early trilobite cephalic limbs. The presence of a fully developed biramous organization in this appendage implies combined roles in locomotion, feeding, and possibly respiration.

Although the interpretations presented here are constrained by a limited sample size, the exceptional three-dimensional preservation afforded by micro-CT scanning provides robust anatomical data that substantially refine previous reconstructions based on compressed material.

##  Supplemental Information

10.7717/peerj.21095/supp-1 Supplemental Information 1 Specimens of *Yunnancephalus yunnanensis* (Mansuy, 1912) studied herein (A) YKLP 17210, dorsal view. (B) YKLP 17211, dorsal view. Scale bars: 2.5 mm.

## Additional Information and Declarations

### Data Availability

The following information was supplied regarding data availability: Original CT-Scan TIFF stacks of the studied specimens of *Y. yunnanensis* can be found online at the following links: YKLP 17210: Liu, Yu; Mai, Huijuan; Collantes, Luis; Hou, Xianguang (2026), “Original CT tiffstack of YKLP 17210 for “Three-dimensionally preserved appendages of the early Cambrian trilobite *Yunnanocephalus yunnanensis*””, Mendeley Data, V1, doi: 10.17632/z93mjmpt8j.1- https://data.mendeley.com/datasets/z93mjmpt8j/1. YKLP 17211: Liu, Yu; Mai, Huijuan; Collantes, Luis; Hou, Xianguang (2026), “Original CT tiffstacks of YKLP 17211 for “Three-dimensionally preserved appendages of the early Cambrian trilobite *Yunnanocephalus yunnanensis*””, Mendeley Data, V1, doi: 10.17632/9wnkb5ncd5.1- https://data.mendeley.com/datasets/9wnkb5ncd5/1.
