# Supplementary figures and images for: Three-dimensionally preserved appendages of the early Cambrian trilobite Yunnanocephalus yunnanensis
Source: PeerJ. 2026 Jun 25;14:e21095. doi: 10.7717/peerj.21095 (PMC13310485; doi:10.7717/peerj.21095)

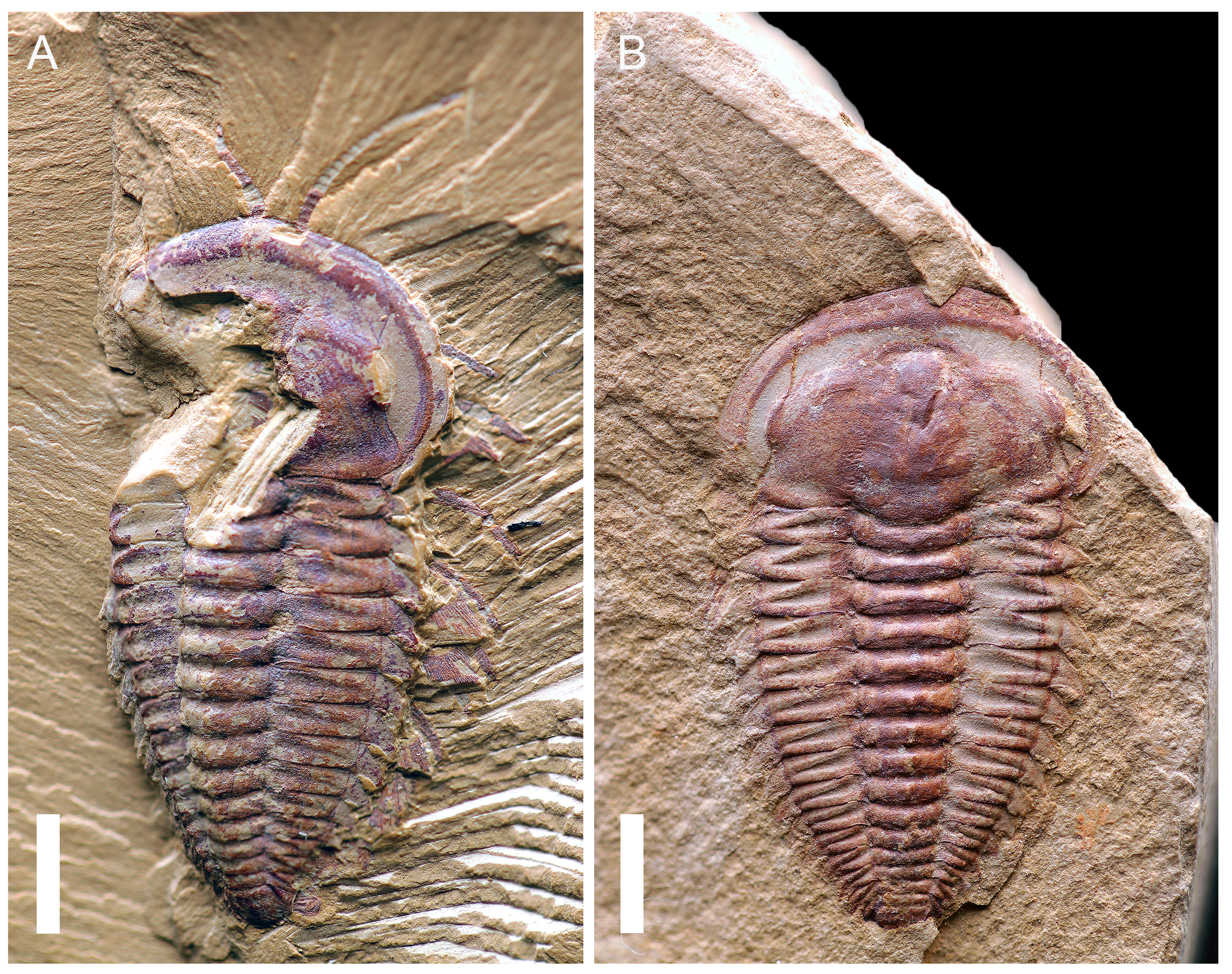

Supplement: Supplemental Information 1 — (A) YKLP 17210, dorsal view. (B) YKLP 17211, dorsal view. Scale bars: 2.5 mm. [file peerj-14-21095-s001.png]
